# Revascularization Techniques for Limb Salvage in Critical Limb Ischemia: A Single Institutional Study From Pakistan

**DOI:** 10.7759/cureus.27900

**Published:** 2022-08-11

**Authors:** Tehreem Kazmi, Faiza H Soomro, Mehwish Ansar

**Affiliations:** 1 General Surgery, Shifa International Hospital Islamabad, Islamabad, PAK; 2 General Surgery, The Dudley Group NHS Foundation Trust, Dudley, GBR; 3 General Surgery, Pakistan Institute of Medical Sciences, Islamabad, PAK

**Keywords:** vascular surgery, limb salvage, endoscopic angioplasty, critical limb ischemia, bypass surgery

## Abstract

Objectives: The study aimed to determine the frequency of successful limb salvage in patients presenting with critical limb ischemia utilizing the available revascularization modalities. This descriptive cross-sectional study was conducted in the Department of General Surgery, Shifa International Hospital, Islamabad, from April 2017 to August 2017.

Methodology: A total of 96 patients with critical limb ischemia requiring urgent surgery for limb salvage were included in our study. Patients who had undergone previous surgeries for limb ischemia involving the same limb, had concurrent venous disease, or suffered from acute limb ischemia were excluded. All patients underwent either endoscopic angioplasty or bypass surgery. All patients were followed up for six months for the success of limb salvage and the requirement for amputation. Data were analyzed by SPSS version 26.0 (Armonk, NY: IBM Corp.).

Results: Our patients had a mean age of 62.03±8.46 years, of whom 63 (65.6%) were men. A total of 47 (49.0%) patients required surgery for a non-healing ulcer, while 49 (51.0%) had resting leg pain. In 55 (57.3%) patients, bypass surgery was performed, while 35 (36.5%) underwent endoscopic angioplasty. The remaining six (6.2%) patients received a combination of both procedures. Limb salvage was successful in 78 (81.3%) patients. There was no difference between outcomes across gender (p=0.122), nor was there any difference in outcome between bypass surgery and endoscopic angioplasty (p=0.665).

Conclusion: Encouraging results can be obtained in treatment of critical limb ischemia if revascularization techniques are utilized prudently in a time-effective manner and individualized to each patient's requirements.

## Introduction

Chronic lower limb ischemia has a global prevalence of approximately 202 million, making it one of the most common causes of presentation to the vascular surgeon [1]. An estimated 25% of these patients will develop critical limb ischemia (CLI) [2]. The Trans-Atlantic Society of Cardiovascular Surgery (TASC) defines critical limb ischemia as a chronic process characterized by the presence of ischemic ulcer necrosis or rest pain, limb-threatening ischemia (necessitating amputation within six months), as well as certain specific ankle and toe pressure thresholds [3].

Management involves early revascularization, which has traditionally been based on open surgical techniques such as surgical bypass grafting, using a vein autograft which was the criterion standard for revascularization for CLI [4], but newer surgical modalities involving endovascular procedures such as percutaneous transluminal angioplasty (PTA), with or without stenting, are becoming increasingly in-vogue due to purportedly lower morbidity and mortality [5]. Of note, traditional open surgical methods are associated with a 15-20% rate of amputation within one year of surgery, while studies researching the newer technique have reported a drop in amputation rates during this time frame [6,7]. However, it is pertinent to note that this benefit has not been noted universally. Studies have reported that, while perioperative morbidity was lower with endovascular techniques, there was a higher requirement for re-surgery, and overall amputation rates were similar or even higher when compared to open surgical techniques [8,9]. Thus the decision on which technique to employ remains the subject of the same debate due to the limited quantity of randomized data available on the subject [10].

Limb salvage with aggressive management using early revascularization remains the first-line treatment for critical limb ischemia in eligible patients. The choice of open surgical versus endoscopic procedure has to be individualized for every patient. Data for limb salvage after any intervention are non-existent in Pakistan, which is especially plagued by a paucity of resources. We conducted this study to determine the limb salvage rate in critical limb ischemia, aiming to establish best practices based on our study results. This is of particular use in current times as the rising epidemic of diabetes mellitus and hypertension contributes to the expansion of the number of patients suffering from critical limb ischemia.

## Materials and methods

This study was conducted from April 2017 to October 2017 in the Department of Surgery, Shifa International Hospital, Islamabad, on 96 patients who gave informed consent. It was designed as a descriptive cross-sectional study, and the patients were selected via non-probability, consecutive sampling. We used the WHO sample size calculator to calculate the sample size keeping an absolute precision (d) of 8.9%, confidence level (1-α) at 95%, and anticipated population proportion (P) of 73.0% [11]. Patients of both genders, ages between 16 years and 75 years, and diagnosed with critical limb ischemia were included. Those who had undergone previous surgeries for limb ischemia involving the same limb had concurrent venous disease or suffered from acute limb ischemia were excluded from the study.

Patients were labeled as suffering from critical limb ischemia if they developed either rest pain of more than 5 out of 10 on a visual analog pain scale or had a non-healing ulcer on the distal lower limb extremity that had lasted for over two weeks and had an ankle brachial pressure index of less than 0.7 and fell into Rutherford categories 4 (rest pain) and 5 (non-healing ulcer with minor tissue loss). CT angiogram was done in all patients, and revascularization modality was decided based on the CT angio findings; short segment occlusion was treated essentially with endoscopic techniques like balloon dilatation and stent placement, while long segment diseases were treated with formal bypass surgeries. Some patients required both modalities based on having simultaneous long and short segment disease.

The endoscopic procedures were performed under local anesthesia with or without sedation, while surgical bypasses were done under general anesthesia after preoperative workup, including echo, to assess cardiac status. Some patients who had long segment disease but were not fit for general anesthesia were also offered endoplastic revascularization.

Postoperatively limbs were assessed mainly clinically by assessing signs and symptoms of ischemia, checking pulse with or without handheld doppler or duplex scan if required. Successful limb salvage was defined as an amputation-free survival of six months, with improvement in the symptoms, i.e., a decrease in pain intensity of at least 50% from the time of presentation and healing of the lower extremity ulcer within one month of surgery.

Data were analyzed using Statistical Package for the Social Sciences version 26.0. Mean and SD was calculated for quantitative variables, specifically age. Qualitative variables like gender, indication for surgery, anatomical side, Rutherford grade, treatment modality used, and whether limb salvage was successful were recorded in frequency and percentage. The Chi-square test was applied to all qualitative variables, while the independent samples t-test was used for quantitative variables to compare the groups. The p-value of ≤0.05 was considered significant.

## Results

Our study consisted of 96 patients, with a mean age of 62.03±8.46 years. There was a preponderance of male patients in our sample, i.e., 63 (65.6%). A total of 47 (49.0%) patients presented with a non-healing ulcer as indication for intervention while the remaining 49 (51.0%) had resting leg pain. The left leg was affected in 51 (53.1%), while 45 (46.9%) had a right leg pathology, while 54 (56.2%) and 42 (43.8%) presented with Rutherford grades 4 and 5 disease, respectively. The pre-procedure characteristics of the patients according to gender are displayed in Table 1.

**Table 1:** Presurgery patient characteristics according to gender.

Variable		Male	Female	p-Value
Gender		63 (65.6%)	33 (34.4%)	-
Age		60.84±8.59	64.30±7.84	0.057
Indications for surgery	Non-healing ulcer	29 (46.0%)	18 (54.5%)	0.428
	Rest pain	34 (54.0%)	15 (45.5%)	
Anatomical side affected	Left	31 (49.2%)	20 (60.6%)	0.288
	Right	32 (50.8%)	13 (39.4%)	
Rutherford grade	Grade 4	38 (60.3%)	16 (48.5%)	0.267
	Grade 5	25 (39.7%)	17 (51.5%)	

Bypass surgery was performed in 55 (57.3%) patients, while 35 (36.5%) underwent endoscopic angioplasty. The remaining six (6.2%) patients received a combination of both procedures. Limb salvage was successful in 78 (81.3%) patients. The surgery and postsurgery results according to gender are shown in Table 2.

**Table 2:** Surgery and postsurgical results according to gender.

Variable		Male	Female	p-Value
Type of surgical procedure	Bypass surgery	35 (55.6%)	20 (60.6%)	0.890
	Endoscopic angioplasty	24 (38.1%)	11 (33.3%)	
	Bypass surgery and endoscopic angioplasty	4 (6.3%)	2 (6.1%)	
Limb salvage success	Yes	54 (85.7%)	24 (72.7%)	0.122
	No	9 (14.3%)	9 (27.3%)	

In addition, we attempted to determine whether bypass surgery was superior to endoscopic angioplasty, or whether a combination would be more effective, in our setting, and found that there was no difference between the procedures with regards to success of limb salvage, as displayed in Table 3.

**Table 3:** Limb salvage success in different types of procedures.

Salvage successful	Yes	No	p-Value
Type of surgical procedure			
Bypass surgery	43 (55.1%)	12 (66.7%)	0.665
Endoscopic angioplasty	30 (38.5%)	5 (27.7%)	
Bypass surgery and endoscopic angioplasty	5 (6.4%)	1 (5.6%)	

## Discussion

Critical limb ischemia is a source of significant morbidity in the elderly, requiring intensive management. Treatment of this disorder requires precise tailoring as each aging patient has their own set of co-morbidities that dictate changes. Our sample had a mean age of 62.03±8.46 years. Dua and Lee reported that peripheral arterial disease had an overall prevalence of 3-10%, which rose dramatically to 15-20% in individuals aged above 70 years, with a corresponding increase in critical limb ischemia [12]. Advanced age, in itself a risk factor for peripheral arterial disease and critical limb ischemia, is associated with a higher incidence of diabetes mellitus and hypertension, which is the reason why these disorders are more common in old age [6].

The majority of cases in our study were male, i.e., 65.6%. This was consistent with McCoach et al., who reported that the population proportion of men in their study was 55.7%. However, women were reported to have a much higher disease grade at the presentation time [13]. Moreover, females have been demonstrated to have a lower chance of successful revascularization and a greater chance of mortality [14,15].

Successful limb salvage was seen in 81.3% of our cases. Of these, 55.1% of patients received bypass surgery, 38.5% received endoscopic angioplasty, while 6.4% a combination of both procedures, and there was no statistical difference between outcomes in our study (p=0.665). Our results were similar to Fu et al., who also concluded that the two procedures were equivalent [16]. Critical limb ischemia appears to develop in approximately 1% of patients suffering from peripheral arterial disease and carries an overall survival rate of 50% at five years and 30% at 10 years [17-19]. Albers et al., in their meta-analysis, revealed that the five-year primary graft patency was 63%, secondary patency was 71%, and the frequency of successful limb salvage post-procedure was 78% in their study figures that were similar to our study [20]. Ghoneim et al. conducted a similar study on higher Rutherford grade diseases ranging from grade IV to VI with advanced aorto-iliac and infra-inguinal lesions and found that primary patency, secondary patency, and successful limb salvage by percutaneous transluminal angioplasty were 77.8, 84.7, and 90.7%, respectively, at two years of follow-up [21].

With regards to outcomes, Conte et al. reported a perioperative mortality rate of 2.7%, and a total of 5.2% of patients who underwent bypass surgery developed graft occlusion [22]. This study followed-up patients for one year and found an 80% patency rate in patients who underwent bypass grafting, 16% of patients were dead, and successful limb salvage was seen in 88% of patients [22]. Chung et al. reported a failure of ulcer healing in 25% of cases at one-year postprocedure, even though the salvage rates were comparable to previously mentioned studies [23]. Conversely, Pua et al. noted that there was a significant improvement in the ulcers of all patients who underwent surgical procedures to restore adequate circulation, i.e., 100% of the ulcers healed, with a limb salvage rate of 78% at one-year follow-up [24]. Lastly, Albers et al. reported a total amputation rate of 11.6% in their analysis, while Goodney et al. reported the same at 12% at one-year follow-up for patients with critical limb ischemia [20,25].

Medical management is considered in patients who are not qualified for surgery and is successful in only 53.6% of patients with critical limb ischemia [11]; furthermore, Thomas et al. reported a slightly higher successful limb salvage rate of 58% with medical management [26]. We believe the conservative management of critical limb ischemia needs further evaluation before concrete proposals can be formulated.

Our study also demonstrated no difference between bypass surgery and endoscopic angioplasty in terms of outcomes, while the occurrence of complications is less well-studied in literature but may be higher with bypass grafting. Overall, limb salvage rates and amputation-free rates appear to be similar.

Limitations

Management of critical limb ischemia is a challenging task and the treatment of this increasingly common malady faces a general dearth of comparative, randomized, and blinded research. We conducted this study in an attempt to widen the knowledge base in literature on the management of this disease, however, some limitations were present. Firstly, our follow-up period was short, and we were unable to monitor the occurrence of long-term complications and outcomes. Second, our study lacked randomization, the decision to undergo bypass grafting or endoscopic angioplasty was individualized based on every patient’s circumstances, co-morbidities, and the type of obstruction and CT findings, which may have introduced some confounding in our results. Lastly, there was limited comparative data available on the subject in international literature, which made a basis for comparison difficult.

## Conclusions

Bypass grafting and endoscopic angioplasty are both beneficial for the treatment of critical limb ischemia with promising results if wisely individualized according to patients’ needs. Further research should focus on the long-term outcomes of both procedures through carefully controlled, randomized, and blinded trials.
